# Novel function of FAXDC2 in megakaryopoiesis

**DOI:** 10.1038/bcj.2016.87

**Published:** 2016-09-30

**Authors:** Q Jin, Y Ren, M Wang, P K Suraneni, D Li, J D Crispino, J Fan, Z Huang

**Affiliations:** 1 College of Life Sciences, Wuhan University, Wuhan, Hubei, People's Republic of China; 2 Feinberg School of Medicine, Northwestern University, Chicago, IL, USA; 3 Department of Hematology, Tongji Hospital, Huazhong University of Science and Technology, Wuhan, Hubei, People's Republic of China; 4 Department of Pediatrics, Zhongnan Hospital of Wuhan University, Wuhan, Hubei, People's Republic of China

## Abstract

FAXDC2 (fatty acid hydroxylase domain containing 2) is a member of the fatty acid hydroxylase superfamily. Given the important role of fatty acids in megakaryocytes, we have studied the role of this gene in the development of this lineage. Here we show that the expression of FAXDC2 is constantly elevated during megakaryocyte maturation. In contrast, FAXDC2 is significantly downregulated in acute myeloid leukemia and acute megakaryoblastic leukemia. Moreover, FAXDC2 overexpression promotes the differentiation of megakaryocytic cell lines and primary cells, whereas its knockdown disrupts their maturation. Mechanism study shows that FAXDC2 overexpression enhances extracellular signal-regulated kinase (ERK) signaling and increases RUNX1 (Runt-related transcription factor 1) expression. FAXDC2 also restores megakaryocytic differentiation in cells exposed to an ERK inhibitor or those expressing a dominant negative form of RUNX1. Finally, FAXDC2 overexpression leads to an increase in sphingolipid GM3 synthase, suggesting a potential role of FAXDC2 in lipid metabolism that increases ERK signaling and facilitates megakaryocyte differentiation. Together, these results show that FAXDC2 plays a novel role in development of megakaryocytes and its dysregulation may contribute to abnormal hematopoietic cell development in leukemia.

## Introduction

Inducing leukemic cells to undergo differentiation has great potential as a therapeutic strategy, Indeed, the combination of all-*trans* retinoic acid and arsenic trioxide induces complete remission in the vast majority of patients with acute promyelocytic leukemia.^1^ Furthermore, a recent study demonstrated that the selective aurora kinase A inhibitor MLN8237 promoted the transition from the proliferative cell cycle to an endomitotic one, resulting in differentiation of acute megakaryoblastic leukemia blasts and of atypical megakaryocytes in primary myelofibrosis.^2, 3^ Unfortunately, not all hematologic malignancies are amenable to differentiation therapy. Therefore, new therapeutic targets and new strategies to eliminate tumor cells are required.

The extracellular signal-regulated kinase (ERK) signaling cascade plays an important role in the regulation of various cellular processes including proliferation, differentiation, development, survival, learning and apoptosis.^4^ ERK signaling activated downstream of thrombopoietin signaling in megakaryoblastic cell lines, primary murine cells, and human progenitor cells also has been shown to be critical for differentiation of hematopoietic progenitors into megakaryocytes.^5^ The function of ERK in 12-*O*-tetradecanoylphorbol-13-acetate (TPA)-induced megakaryocytic differentiation of K562 cells has been extensively studied.^6^ Furthermore, a recent report demonstrated that some genes promote TPA-induced megakaryocytic differentiation of K562 cells through ERK signaling,^7, 8, 9^ suggesting that this pathway is one of the key regulators that may be subjected to the other molecules during megakaryocytic differentiation.

RUNX1 (Runt-related transcription factor 1) is one of the downstream effector of ERK signaling.^8^ RUNX1 participates in a variety of normal hematopoiesis including megakaryopoiesis and maturation of T and B lymphocytes, while mutation of runx1 may lead to the increasing risk of acute myeloid leukemia (AML).^10^ *In vivo*, inducible knockout of RUNX1 in bone marrow showed inhibition of megakaryocytic maturation as evidenced by reduced polyploidization and defective development of demarcation membranes in the megakaryocytes.^11^ Moreover, the chimeric gene product of t(8;21), RUNX1-RUNX1T1 (also known as RUNX1-ETO or AML1-MTG8), discovered in human leukemia has been demonstrated to repress the function of normal RUNX1 in hematopoiesis, suggesting that loss of RUNX1 function is a common feature of leukemogenesis by these mutants.^12^ Thus, inducing forced megakaryocytic differentiation of leukemic cells by enhancing RUNX1 expression may possibly be an efficient way to develop novel antileukemia therapy.

In our studies to identify novel genes that participate in normal hematopoiesis and leukemia, we noticed that the novel gene *FAXDC2* (*fatty acid hydroxylase domain containing 2*) was downregulated in AML patients and increased in its expression during TPA-induced megakaryocytic differentiation of K562 cells. There have been few reports of the role of FAXDC2, and thus little is known about its function. In this study, we revealed that FAXDC2 promoted megakaryocytic differentiation in K562 cells and overexpressing human FAXDC2 in murine bone marrow progenitors resulted in increased megakaryocytic differentiation. Furthermore, enhancing of ERK signaling and its downstream effector RUNX1 participated in the mechanism underlying the role of FAXDC2 in megakaryocytic differentiation. Thus, we have identified the function of FAXDC2, a novel gene that promotes megakaryocytic maturation and suggests that it may have potential value as differentiation therapy.

## Materials and methods

### Cell culture

K562 and HEL cells were maintained in RPMI-1640 medium and HEK293T cells were cultured in Dulbecco's modified Eagle's medium (Gibco BRL, Grand Island, NY, USA), both of which were supplemented with 10% fetal bovine serum (Thermo Scientific HyClone, Logan, UT, USA) and penicillin/streptomycin. Primary mouse megakaryocytes were cultured and maintained as described previously.^13, 14^ C57BL/6 mice were maintained in micro isolator housing within a barrier facility. The Animal Care and Use Committees of Wuhan University approved all animal studies. Peripheral blood samples were obtained from newly diagnosed AML patients and healthy donors. All experiments involving in human blood samples were approved by the Medical Ethics Committee of Tongji Hospital of Huazhong Technology University. Human megakaryocyte culture experiments were performed by culturing CD34+ cells (purchased from Fred Hutchinson Cancer Research Center, Seattle, WA, USA) in StemSpam SFEM media (Stemcell Technologies, Vancouver, BC, Canada) supplemented with penicillin/ streptomycin, lipids (40 mg/ml), stem cell factor (100 ng/ml) and thrombopoietin (50 ng/ml) for 9 days.

### Lentivirus and retrovirus infection

FAXDC2 overexpression and knockdown cell lines were generated by lentiviral transduction as previously described.^15^ Lentiviral vector (pHAGE) and (pLKO.1) were used for overexpression and knockdown, respectively. Vectors carried either puromycin or green fluorescent protein for selection as indicated. Short hairpin RNA (shRNA) sequences for FAXDC2 knockdown were designed by online shRNA search tool (http://www.invivogen.com/sirnawizard/design_advanced.php). The sequences of shRNA specific for human FAXDC2 used in this study were as following: shFAXDC2#1: 5′-GAGCTTCAATGGGCTTCTATT-3′ shFAXDC2#2: 5′-GAGGAAGTCTTGTTCTACTAT-3′.

### Flow cytometry analysis

K562 cells were induced to differentiate with TPA (10 nM) for hours as indicated. The treated cells were washed with phosphate-buffered saline and stained with phycoerythrin-conjugated anti-CD61 antibody or anti-CD41 antibody (BD Biosciences, San Jose, CA, USA). Differentiation of primary mouse megakaryocytes was performed by staining cells with fluorescently labeled anti-CD41 (cat. no. 17-0411, eBioscience, San Diego, CA, USA) or anti-CD42 (cat. no. 12-0421-82, eBioscience) antibodies or by staining DNA with 4,6-diamidinio-2-phenylindole (DAPI, 1 mg/ml). FACS data were acquired with a FACSAria instrument (BD Biosciences, Mountain View, CA, USA) and analyzed with FlowJo software (TreeStar, Ashland, OR, USA). All flow cytometry data depict the representative results from three independent experiments with duplicates.

### RNA preparation and quantitative real-time PCR

Total RNA was extracted with Trizol and complementary DNA was prepared with reverse transcriptase by standard procedure following instructions of the manufacturer (Invitrogen, Grand Island, NY, USA). Quantitative PCR was performed under the following conditions: hot start at 95 ℃ for 15 min followed by 95 ℃ for 30 s, 63 ℃ for 30 s and 72 ℃ for 30 s for 40 cycles. The relative quantities of real-time PCR products were determined using the comparative ΔΔCT method. Primer sequences are available upon request.

### Immunoblotting and antibodies

Immunoblotting was performed as previously described.^9^ Antibodies for HSC70 (cat. no. SC-7298), p-ERK (cat. no. SC-7383) and ERK (cat. no. SC-514302) were purchased from Santa Cruz Biotechnology (Santa Cruz, CA, USA). Antibodies against FLAG (cat. no. F3165) were purchased from Sigma (St Louis, MO, USA). Antibodies for HA (cat. no. 66006-1-lg) and FAXDC2 (cat. no. 22046-1-AP) were purchased from Proteintech (Chicago, IL, USA).

### Fluorescence staining and Wright-Giemsa staining

The 293T cells were grown on glass coverslips overnight, transfected with FAXDC2 lentivirus and fixed after 48 h with 4% paraformaldehyde in phosphate-buffered saline for 25 min at room temperature. Fixed cells were then permeabilized with phosphate-buffered saline/0.1% Tween-20 for 5 min and blocked with 3% bovine serum albumin in phosphate-buffered saline for 1 h. Cells were then incubated in Anti-Flag M2-Cy3-conjugated antibody (1:200; Sigma A9594) and DAPI for 1 h, washed and the coverslips were mounted with the Vectashield mounting medium (Vector Laboratories, Burlingame, CA, USA). Fluorescent images were obtained using the Nikon A1R+ confocal microscope (Nikon, Tokyo, Japan) under a 63 × Plan-Apochromatic oil immersion lens. Wright-Giemsa staining was performed following the manual from the supplier (Sigma). Cell morphology was observed by light microscopy.

### Statistics

All statistical analyses were performed using Student's *t-*test (two tailed, unpaired). A *P-*value of ⩽0.05 was considered significant.

## Results

### FAXDC2 is upregulated during megakaryocytic maturation

**Figure 1:**
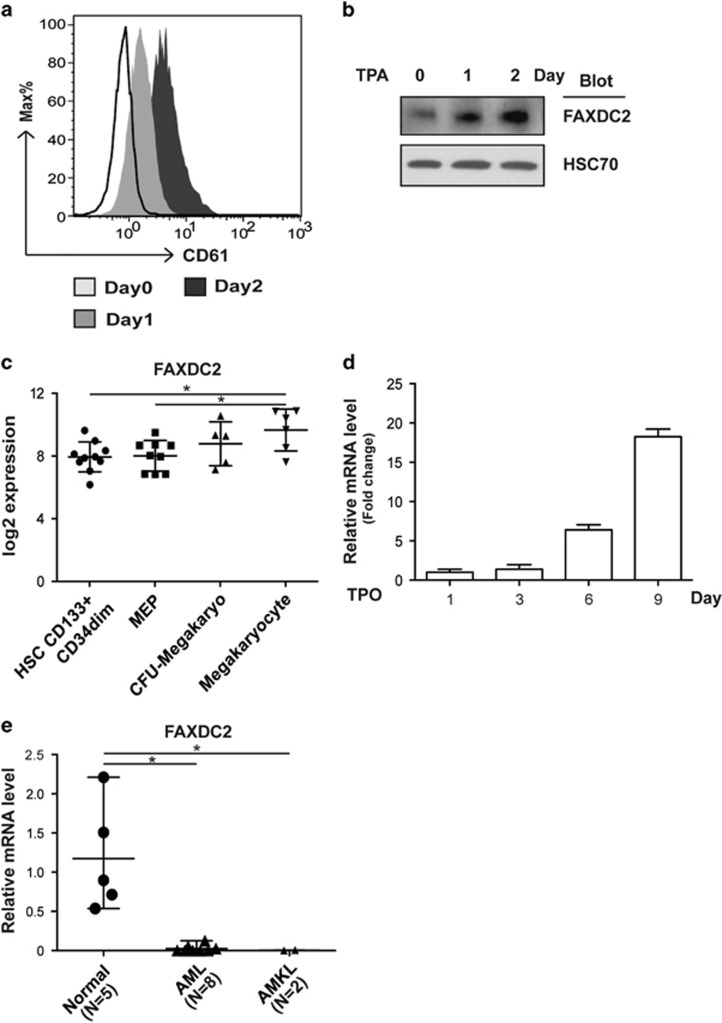
FAXDC2 is upregulated during megakaryocytic maturation and downregulated in AMKL. (**a**) K562 cells were treated with TPA for days as indicated. Megakaryocytic differentiation of K562 cells was measured by staining cells with an anti-CD61-PE antibody and analyzed by flow cytometry. (**b**) K562 cells treated with TPA for times as indicated to measure FAXDC2 expression at protein level by western blot. HSC70 serves as a control. (**c**) The expression of FAXDC2 mRNA in hematopoietic stem cell CD133+CD34dim, megakaryo-erythroid progenitor, colony forming unit-megakaryocyte and megakaryocyte were analyzed and presented as log2 expression. Expression data were obtained from online Bloodspot database (http://servers.binf.ku.dk/bloodspot/?gene=C5orf4&dataset=DMAP). (**d**) Human CD34+ cells were cultured in the presence of 10 ng/ml thrombopoietin (TPO) for consecutive 9 days. The expression of FAXDC2 was detected by quantitative real-time PCR (RT-PCR). (**e**) Quantitative RT-PCR analysis of FAXDC2 in mononuclear cells (MNCs) from healthy donors (Normal, *N=*5), AML patients (*N=*8) and AMKL patients (*N=*2). The expression of FAXDC2 was normalized to GAPDH and presented as relative mRNA level. *P<0.05 compared with control.

A previous report has described the importance of lipid biosynthesis in megakaryocytic differentiation.^16^ Given that FAXDC2 is a member of the fatty acid hydroxylase superfamily, FAXDC2 might play a role in this lineage. In support of this, FAXDC2 expression was shown to be significantly elevated in K562 cells undergoing megakaryocytic differentiation induced by TPA (Figures 1a and b). Our further analysis of an online microarray database of normal human hematopoiesis also revealed that the expression of FAXDC2 was remarkably upregulated as cells committed to and matured as megakaryocytes, with an increase from hematopoietic stem cells (CD133+CD34dim) to megakaryo-erythroid progenitor to colony forming unit megakaryocytes and finally to mature megakaryocytes (Figure 1c).^17^ More importantly, constant upregulation of FAXDC2 was observed in CD34+ cells induced to megakaryocytic differentiation over 9 days (Figure 1d). In sharp contrast, FAXDC2 expression was significantly downregulated in peripheral blood cells from AML and acute megakaryoblastic leukemia (AMKL) patients (Figure 1e). Together, these observations suggest that FAXDC2 participates in megakaryocytic differentiation and its dysregulation may contribute to abnormal hematopoietic cell development in leukemia.

### FAXDC2 reinforces TPA-induced megakaryocytic differentiation

**Figure 2:**
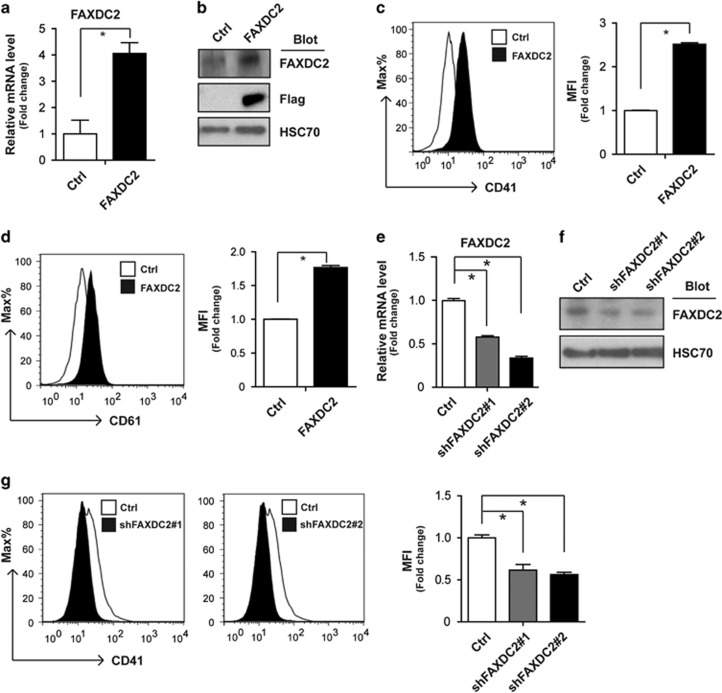
FAXDC2 reinforces TPA-induced megakaryocytic differentiation. (**a**) K562 cells were transduced with a control lentiviral vector (Ctrl) or a FAXDC2-expressing lentiviral vector. The expression of FAXDC2 in the resultant cells was confirmed by quantitative real-time PCR (RT-PCR) at the mRNA level. (**b**) The endogenous or exogenous (Flag-tagged) FAXDC2 protein in the resultant cells was measured by western blot with anti-FAXDC2 or anti-Flag. HSC70 served as a control. (**c**) Control cells or FAXDC2-overexpressing cells were treated with TPA (10 nM) for 2 days. CD41 expression was measured by staining cells with phycoerythrin (PE)-conjugated anti-CD41 antibody and analyzed by flow cytometry (histogram, left panel). Bar graph (right panel) was the statistics of mean fluorescence intensity (MFI) of flow cytometry analysis. (**d**) The expression of CD61 in the resultant cells was also measured and analyzed (histogram, left panel). Bar graph (right panel) was the statistics of left panel. (**e**) K562 cells were transduced with control lentiviral vector (Ctrl) or lentiviral vectors expressing two shRNAs specific for human FAXDC2 (shFAXDC2#1, shFAXDC2#2). The endogenous of FAXDC2 in resultant cells was measured by quantitative RT-PCR at the mRNA level. (**f**) The knockdown of endogenous FAXDC2 protein was confirmed by western blot with anti-FAXDC2. (**g**) The FAXDC2 knockdown cells (shFAXDC2#1 and shFAXDC2#2) and control cells (Ctrl) were treated with TPA for 2 days. The expression of CD41 in the resultant cells was measured (histogram, left panel). Bar graph (right panel) was the statistics of left panel. All histograms were representative results of three independent experiments (duplicates) with similar results. *P<0.05 compared with control.

In an effort to identify the function of FAXDC2 in megakaryocytic differentiation, we first overexpressed FAXDC2 in K562 cells by lentiviral transduction. The extent of overexpression was confirmed by real-time PCR and western blot (Figures 2a and b). Overexpression of FAXDC2 alone did not induce megakaryocytic differentiation (Supplementary Figures S1A and B). However, ectopic expression of FAXDC2 significantly enhanced TPA-mediated megakaryocytic maturation, with a striking enhancement of CD41 and CD61 expression (Figures 2c and d). Furthermore, HEL cells that overexpressed FAXDC2 also exhibited increased megakaryocytic differentiation with TPA treatment (Supplementary Figures S2A and B). To further confirm the function of FAXDC2, we knocked down FAXDC2 with two shRNAs (shFAXDC2#1 and shFAXDC2#2) in K562 cells by lentiviral transduction. FAXDC2 knockdown was confirmed by real-time PCR and western blot (Figures 2e and f). Again, FAXDC2 knockdown did not affect differentiation (Supplementary Figure S3A). In accordance with previous observations, FAXDC2 knockdown impeded TPA-induced megakaryocytic differentiation, as evidenced by reduced CD41 expression (Figure 2g). Together, these results demonstrate that FAXDC2 enhances megakaryocytic differentiation.

### FAXDC2 enhances ERK phosphorylation and upregulates RUNX1 in TPA-induced K562 cells

**Figure 3:**
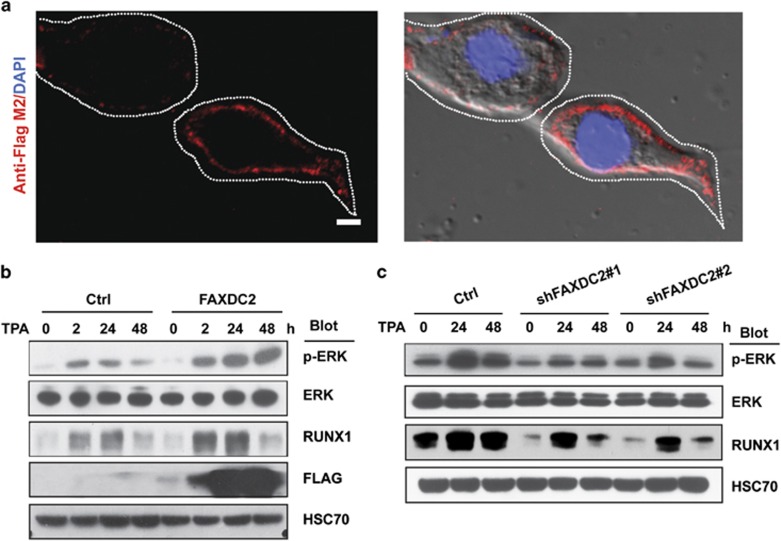
FAXDC2 enhances ERK phosphorylation and upregulates RUNX1 in TPA-induced K562 cells. (**a**) Cytosolic localization (left panel) of FAXDC2 was observed in 293T cells overexpressing Flag-Tagged FAXDC2 protein (red). Right panel depicts merged image of FAXDC2 together with nuclear DAPI staining (blue). Scale bar: 75 μm. (**b**) K562 cells transduced with control (Ctrl) or FAXDC2-overexpressing lentiviral vector were induced with TPA for indicated hours. The phosphorylation of ERK (p-ERK), ERK, Flag and the expression of RUNX1 were measured. HSC70 served as control. (**c**) Control (Ctrl) or FAXDC2 knockdown (shRNA#1 and shRNA#2) K562 cells were stimulated with TPA for hours as indicated. The phosphorylation of ERK (p-ERK), ERK and the expression of RUNX1 were measured by western blot.

To investigate the mechanism by which FAXDC2 contributes to megakaryocytic differentiation, we performed several experiments. First, we overexpressed FAXDC2 in 293T cells and examined its localization by immunofluorescence. We observed that FAXDC2 was primarily expressed in the cytoplasm (Figure 3a). Next, as TPA promotes megakaryopoiesis by modulating cell signaling, notably the ERK pathway, as well as by promoting the expression of the critical megakaryocyte regulator RUNX1,^9^ we investigated whether FAXDC2 participated in this process. Of note, overexpression of FAXDC2 increased expression of RUNX1 and also enhanced ERK signaling (Figure 3c). In contrast, knockdown of FAXDC2 reduced p-ERK and RUNX1 expression (Figure 3d). Taken together, these results suggest that FAXDC2 enhances p-ERK signaling and the subsequent expression of its downstream effector, RUNX1.

### FAXDC2 enhances TPA-induced megakaryocytic differentiation through ERK and RUNX1 in K562 cells

**Figure 4:**
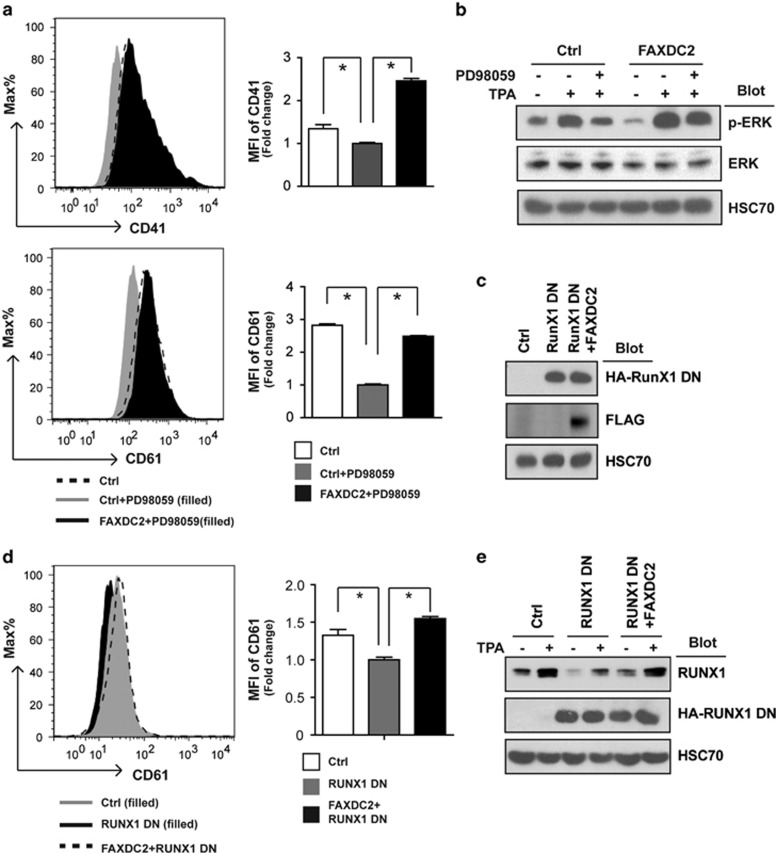
FAXDC2 enhances TPA-induced megakaryocytic differentiation through ERK and RUNX1 in K562 cells. (**a**) Control (Ctrl) or FAXDC2-overexpressing K562 cells were pretreated with (+) or without (−) PD98059 for 2 h and then stimulated by TPA for 2 days. The resultant cells were stained with phycoerythrin (PE)-conjugated anti-CD61 antibody or anti-CD41 antibody and analyzed by flow cytometry (left panel). Bar graph (right panel) was the statistics of left panel. (**b**) Control (Ctrl) or FAXDC2-overexpressing K562 cells were treated (+) or without (−) PD98059 and stimulated by TPA for 2 days. Cells were collected to detect p-ERK by western blot. HSC70 served as a control. (**c**) K562 cells were transduced with pHAGE and pMSCV (Ctrl), pHAGE plus pMSCV RUNX1DN (RUNX1DN) and pHAGE FAXDC2 plus pMSCV RUNX1DN (FAXDC2+RUNX1 DN). HA-tagged RUNX1DN and Flag-tagged FAXDC2 were measured by western bolt. HSC70 served as control. (**d**) The resultant cells stimulated by TPA for 2 days were stained with PE-conjugated anti-CD61 antibody and analyzed by flow cytometry (left panel). Bar graph (right panel) was the statistics of left panel. (**e**) The resultant cells treated with (+) or without (−) TPA were harvested to detect the endogenous RUNX1 and HA-tagged RUNX1DN by western blot. HSC70 served as a control. All histograms were representative results of three independent experiments (duplicates) with similar results. *P<0.05 compared with control.

The critical role of ERK signaling and its downstream effector, RUNX1, has been well studied in megakaryocytic differentiation. To determine the relationship between FAXDC2 and ERK signaling, we treated cells with a specific ERK inhibitor, PD98059, and measured the effect on megakaryopoiesis. Treatment of cells with PD98059 (Ctrl+PD98059) significantly restrained megakaryocytic differentiation, as evidenced by reduced CD41 and CD61 expression compared with the control cells. Of note, ectopic expression of FAXDC2 restored CD41 and CD61 expression to cells treated with the inhibitor (FAXDC2+PD98059) (Figure 4a). This increase was associated with rescue of p-ERK levels (Figure 4b). These findings demonstrate that FAXDC2 may promote TPA-induced megakaryocytic differentiation by enhancing ERK signaling.

ERK activation leads to upregulation of RUNX1. To study the extent to which RUNX1 mediated the link between FAXDC2 and megakaryocyte differentiation, we suppressed endogenous RUNX1 by overexpressing a dominant negative variant (RUNX1 DN) (Figure 4c). As expected, RUNX1 DN restrained megakaryocytic differentiation as evidenced by decreased CD41 expression. Of note, overexpression of FAXDC2 (FAXDC2+RUNX1 DN) restored the expression of the endogenous RUNX1 and rescued megakaryocyte differentiation (Figures 4d and e). Therefore, our observations suggest that FAXDC2 enhances megakaryocytic differentiation by strengthening ERK signaling and expression of RUNX1, a vital transcriptional factor of megakaryocytic differentiation.

### FAXDC2 expedites megakaryopoiesis in murine bone marrow cells

**Figure 5:**
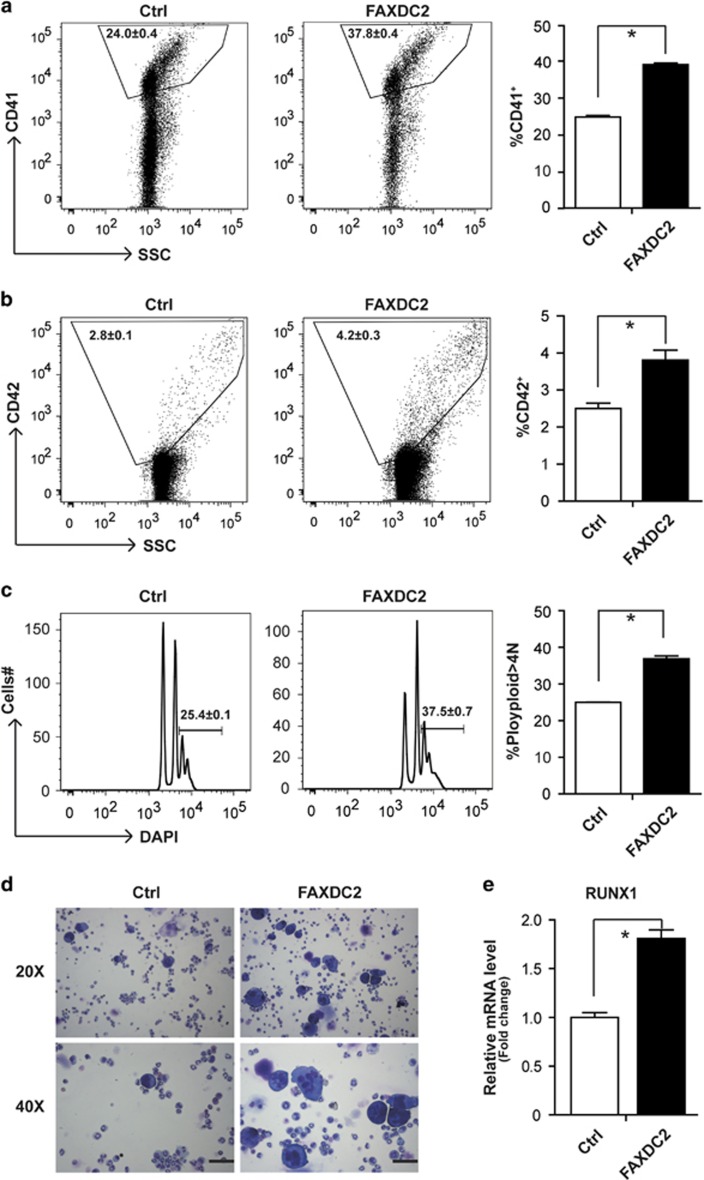
FAXDC2 expedites megakaryopoiesis in murine bone marrow cells. (**a**) c-kit-positive progenitor cells isolated from primary murine bone marrow cells were transduced with control lentiviral vector (Ctrl) or FAXDC2-overexpressing (FAXDC2) vector to undergo megakaryocytic differentiation with thrombopoietin (TPO) for 6 days. The resultant cells were stained with phycoerythrin (PE)-CY7-conjugated anti-CD41 antibody and analyzed by flow cytometry (left panel). Bar graph (right panel) was the statistics of left panel. (**b**) The resultant cells were stained with PE-conjugated anti CD42 antibody and analyzed by flow cytometry (left panel). Bar graph (right panel) was the statistics of left panel. (**c**) The resultant cells were stained with anti-CD41-PE-CY7 and DAPI. The CD41^+^cells were gated for analysis of DNA content. The DNA content that is >4N is represented (left panel). Bar graph (right panel) was the statistics of left panel. (**d**) The control (Ctrl) or FAXDC2-overexpressing (FAXDC2) cells were harvested for Wright-Giemsa stains. The stained cells were photographed under microscopy at the bright view of the microscope (magnification × 20 or magnification × 40). Scale Bar: 100 μm. (**e**) The control (Ctrl) or FAXDC2-overexpressing (FAXDC2) cells were harvested for quantitative real-time PCR (RT-PCR) at mRNA level. The expression of RUNX1 was measured. *P<0.05 compared with control.

To further verify the role of FAXDC2 in megakaryopoiesis, we performed similar studies in primary mouse hematopoietic cells. We isolated c-kit-positive cells from primary murine bone marrow, transduced them with control or FAXDC2-overexpressing (FAXDC2) viruses and examined megakaryocytic differentiation. Ectopic expression of FAXDC2 significantly expedited megakaryopoiesis, as determined by increased CD41 and CD42 expression as well as polyploidization of CD41+ cells (Figures 5a–c). Enhancement of differentiation was also detected by analysis of Wright-Giemsa-stained cytospins (Figure 5d). Moreover, consistent with the K562 cell data, increased expression of RUNX1 was also observed upon ectopic expression of FAXDC2 (Figure 5e). These observations confirm that FAXDC2 plays a positive role in megakaryopoiesis.

## Discussion

Megakaryocyte differentiation is orchestrated by transcription factors and signaling that form a widespread regulation of network associated with other molecules. Aberrant megakaryopoiesis leading to indefinite proliferation of megakaryoblasts is a hallmark of AMKL. Further exploration of the mechanism underlying megakaryocyte proliferation/maturation is critical for the development of potential new therapy for AMKL. In our studies, we observed that FAXDC2, a novel gene without a well-described function, was significantly downregulated in AML and AMKL. Function study demonstrated that FAXDC2 promoted megakaryocytic differentiation by enhancing ERK signaling that subsequently upregulated RUNX1. RUNX1 has a critical role in normal and malignant hematopoiesis and megakaryocyte development.^18^ The loss of RUNX1 leads to downregulation of p19 and accelerates the development of MLL-ENL leukemia in mice because of the enhanced proliferation of leukemic cells.^19^ Our study demonstrates that the identification of novel players that potentially modulate leukemia cell proliferation as well as restore the intrinsic differentiation machinery may be helpful for the development of new therapeutic methods.

FAXDC2 bears a fatty acid hydroxylase domain and is related to the fatty acid hydroxylase superfamily including six genes. One of the most prominent genes in this family is FAXDC1 that encodes a protein that catalyzes the synthesis of 2-hydroxysphingolipids, a subset of sphingolipids containing 2-hydroxy fatty acids.^20^ Sphingolipids function in many cellular processes and one of the sphingolipids, ganglioside GM3 (sialosyllactosylceramide), has been demonstrated to induce megakaryocytic differentiation of K562 cells.^21^ GM3 is a sialic acid-containing glycosphingolipid that is expressed at the cell surface and involved in modulation of cell signaling including mitogen-activated protein kinase.^22, 23, 24^ Furthermore, silencing of sialidase Neu3 triggered megakaryocytic differentiation of K562 cells through the increase of ganglioside GM3 resulting in the enhancement of ERK signaling.^25^ Given that FAXDC2 is predicted to participate in lipid oxidization, we examined whether FAXDC2 expression elevated ganglioside GM3 synthase. We found that the mRNA level of GM3 synthase was increased upon FAXDC2 expression (Supplementary Figure S4A), suggesting that FAXDC2 may modulate ERK signaling by regulating the synthesis of sphingolipids such as GM3 to promote megakaryocytic differentiation.

Taken together, we have identified a novel gene, *FAXDC2*, that functions to promote megakaryocytic differentiation through ERK signaling and RUNX1. Our findings shed light on the mechanism of novel genes that are involved in normal and malignant hematopoiesis.
