# Supplementary material for: Novel function of FAXDC2 in megakaryopoiesis
Source: Blood Cancer J. 2016 Sep 30;6(9):e478–. doi: 10.1038/bcj.2016.87 (PMC5056977; doi:10.1038/bcj.2016.87)
Supplement: Supplementary Information [file bcj201687x1.docx]

**Supplementary Information**

# Novel function of FAXDC2 in Megakaryopoiesis

Qi Jin, Yi Ren, Min Wang, Praveen Kumar Suraneni, Dengju Li, John D Crispino, Zan Huang

Supplementary Figures: 4

**
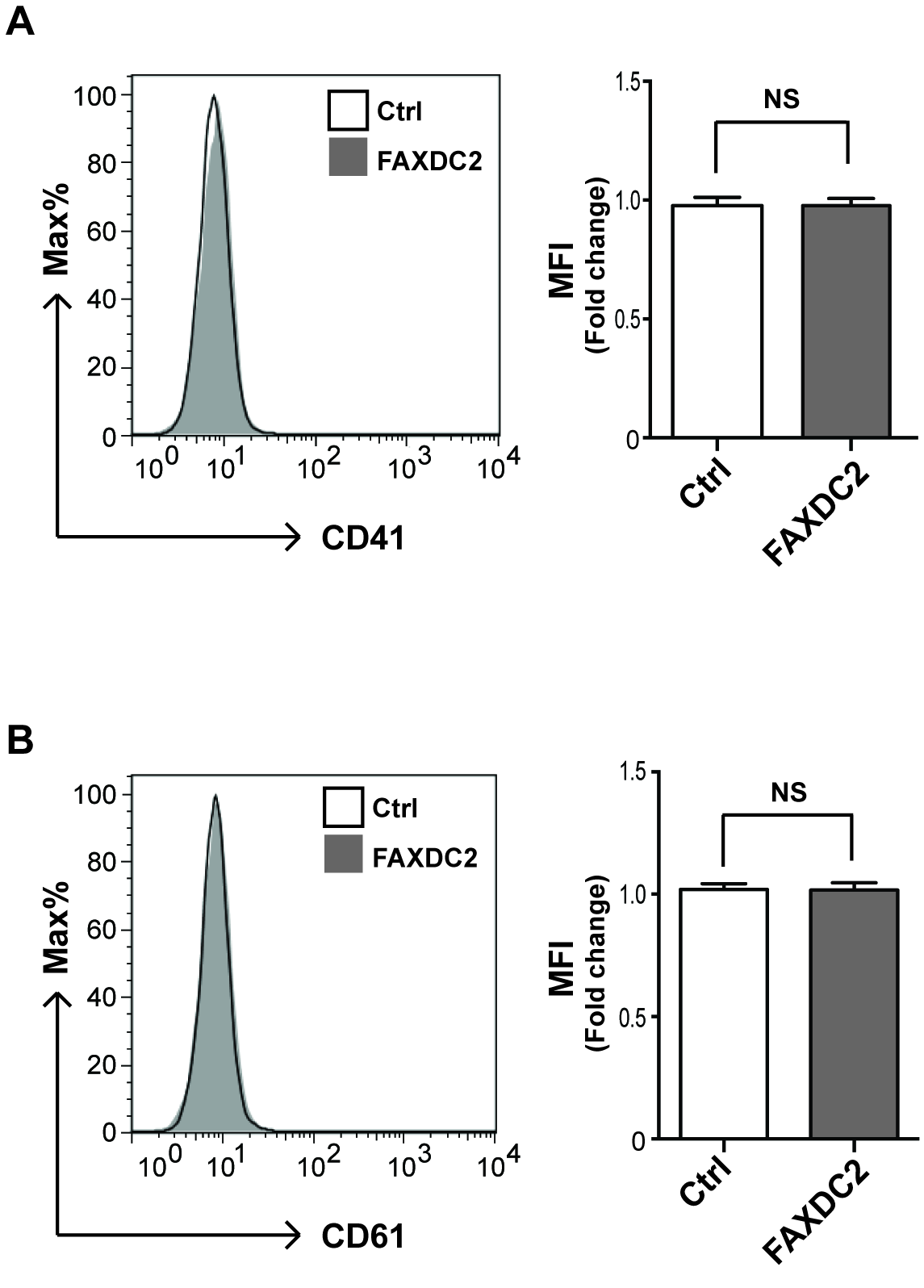
**

**Figure S1** Ectopic expression of FAXDC2 does not induce megakaryocytic differentiation at rest state (**A**) K562 cells were transduced with control lentiviral vector (Ctrl) or FAXDC2-overexpressing (FAXDC2) vector. The control (Ctrl) or FAXDC2-overexpressing (FAXDC2) cells were stained with CD41 for FACS. Bar graph (right panel) was the statistics of left panel. (**B**) The expression of CD61 in the resultant cells was also measured and analyzed (histogram, left panel). Bar graph (right panel) was the statistics of left panel.


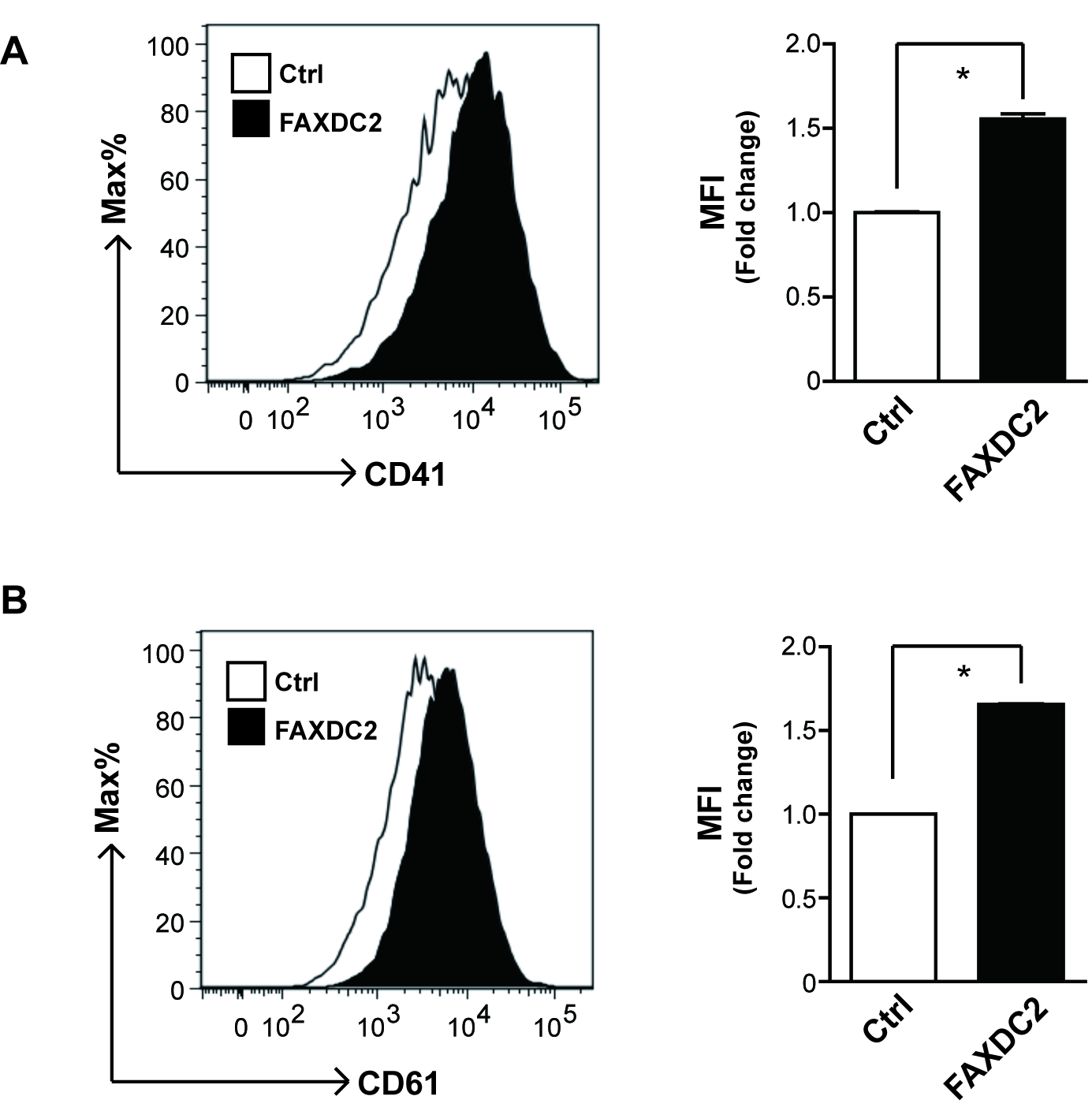


**Figure S2** FAXDC2 promotes megakaryocytic differentiation in HEL cells. (**A**) HEL cells were transduced with control lentiviral vector (Ctrl) or FAXDC2-overexpressing (FAXDC2) vector. The control (Ctrl) or FAXDC2-overexpressing (FAXDC2) cells treated with TPA for 2 days were stained with CD41 for FACS. Bar graph (right panel) was the statistics of left panel. (**B**) The expression of CD61 in the resultant cells was also measured and analyzed (histogram, left panel). Bar graph (right panel) was the statistics of left panel. *Indicates p <0.05 compared with control.


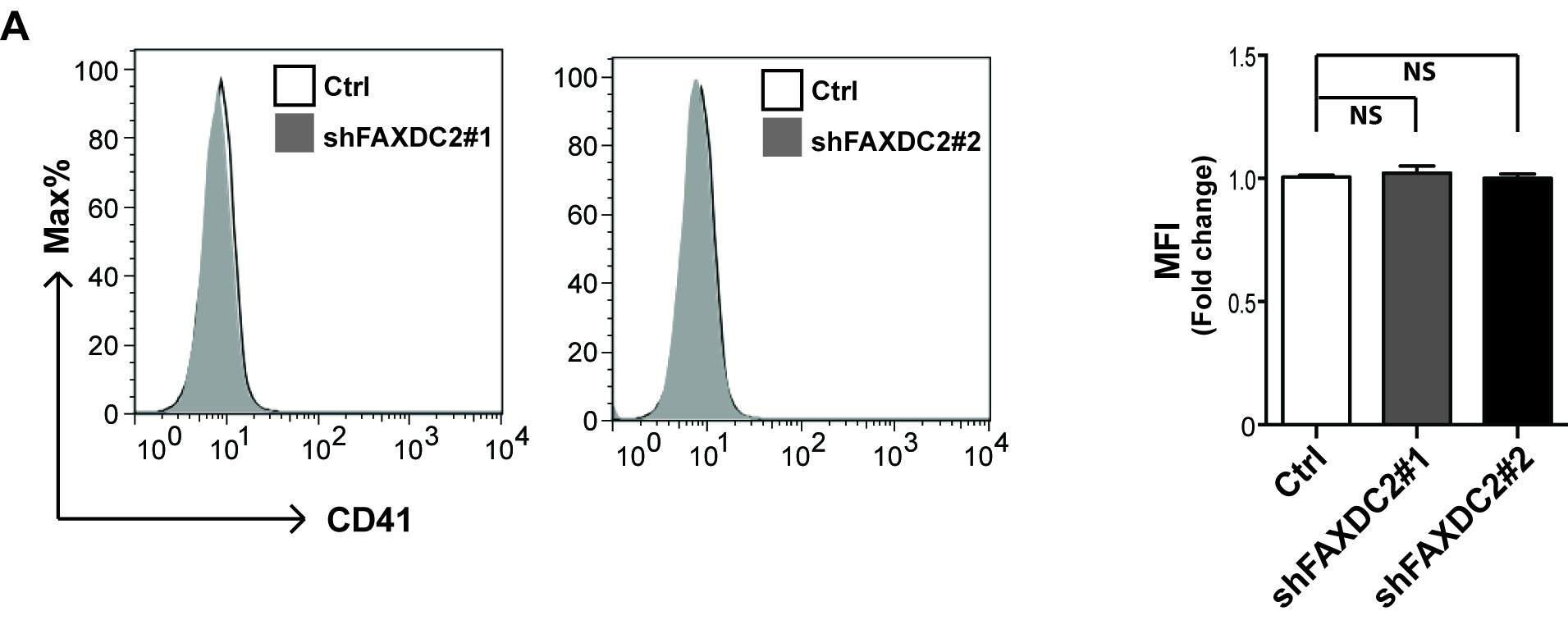


**Figure S3** Knockdown of FAXDC2 does not affect megakaryocytic differentiation at rest state. (**A**) K562 cells were transduced with control lentiviral vector (Ctrl) or lentiviral vectors expressing two shRNAs specific for human FAXDC2 (shFAXDC2#1, shFAXDC2#2). The expression of CD41 in the resultant cells was measured (histogram, left panel). Bar graph (right panel) was the statistics of left panel.


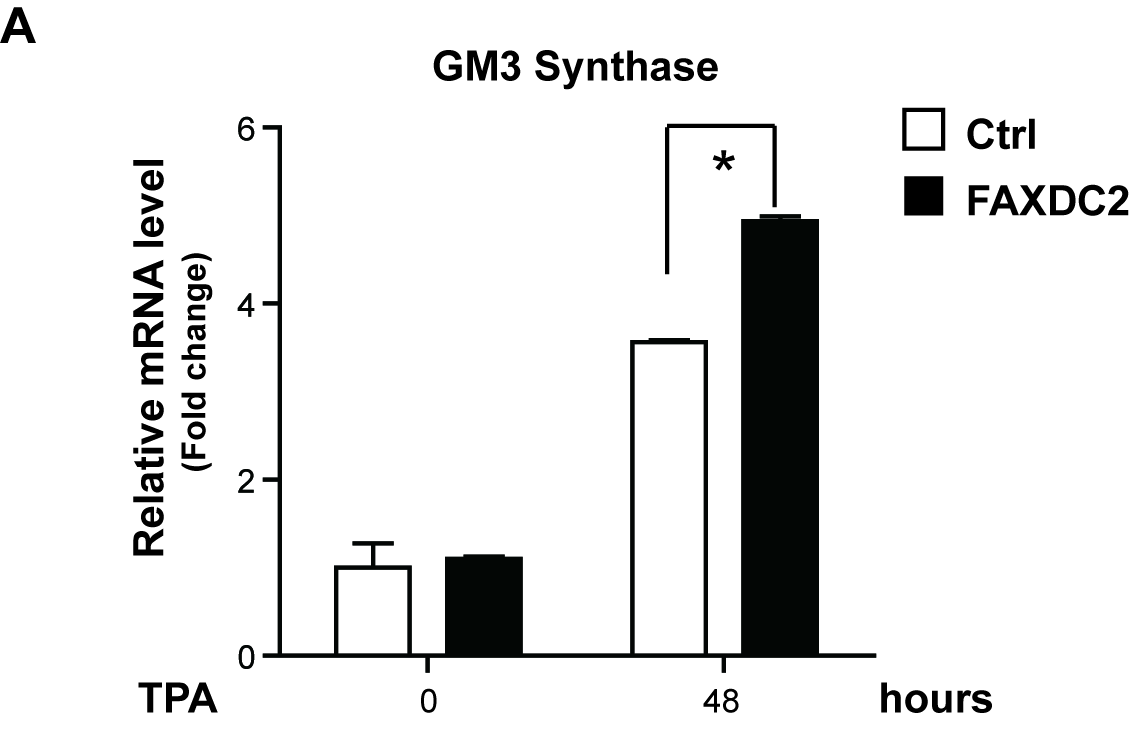


**Figure S4** FAXDC2 upregulates GM3 synthase. (**A**) K562 cells transduced with control (Ctrl) or FAXDC2-overexpressing (FAXDC2) were treated with TPA for indicated times respectively. The resultant cells were collected for measuring GM3 synthase at mRNA level by quantitative RT-PCR. *Indicates p <0.05 compared with control.
